# Specifying and reporting complex behaviour change interventions: the need for a scientific method

**DOI:** 10.1186/1748-5908-4-40

**Published:** 2009-07-16

**Authors:** Susan Michie, Dean Fixsen, Jeremy M Grimshaw, Martin P Eccles

**Affiliations:** 1 Centre for Outcomes Research and Effectiveness, Department of Clinical, Educational and Health Psychology, University College London, 1-19 Torrington Place, London, WC1E 7HB, UK; 2 FPG Child Development Institute, University of North Carolina–Chapel Hill, 517 S Greensboro Street, Carrboro, NC 27510, USA; 3 Clinical Epidemiology Program, Ottawa Health Research Institute, 1053 Carling Avenue, Room 2-017, Admin Building, University of Ottawa, Ottawa, ON, K1N 6N5, Canada; 4 Institute of Health and Society, Newcastle University, 21 Claremont Place, Newcastle upon Tyne, NE2 4AA, UK

## Abstract

Complex behaviour change interventions are not well described; when they are described, the terminology used is inconsistent. This constrains scientific replication, and limits the subsequent introduction of successful interventions. *Implementation Science *is introducing a policy of initially encouraging and subsequently requiring the scientific reporting of complex behaviour change interventions.

## The current state of affairs

Progress in tackling today's major health and healthcare problems requires changes in behaviour [1,2]. Population health can be improved by changing behaviour in those who are at risk from ill health, in those with a chronic or acute illness, and in health professionals and others responsible for delivering effective, evidence-based public health and healthcare. In the field of implementation research, thousands of studies have developed and evaluated interventions aimed at bringing the behavior of healthcare professionals into line with evidence-based practice. Systematic reviews of behaviour change interventions have tended to find modest and worthwhile effects but no clear pattern of results favouring any one particular method. Where effects are found, it is often unclear what behaviour change processes are responsible for observed changes. If effective interventions to change behaviours are to be delivered to influence outcomes at population, community, organisational or individual levels [3], the field must produce greater clarity about the functional components of those interventions. These should then be matched to population, setting, and other contextual characteristics [4].

## What is the problem?

### Interventions aren't described

Few published intervention evaluations refer to formal documentation describing the content and delivery of an intervention and are seldom reported by researchers or practitioners in enough detail to replicate them [5,6]. Reviews of nearly 1,000 behaviour change outcome studies [7-10] found that interventions were described in detail in only 5% to 30% of the experimental studies. Even when the intervention was documented (*e.g*., a detailed manual was available), only a few investigators actually measured the presence or strength of the intervention in practice, and fewer still included such measures in the analyses of the results. Thus, we are often left knowing very little about the details of an intervention or the functional relationship between the components of the intervention and outcomes. Knowing the details and functional relationships are critical to any future introduction and scale-up of effective interventions. This knowledge helps to inform what to teach to new practitioners, how to transform or reorganise healthcare processes, and what to include in the assessment of practitioner performance (fidelity measures)–all key features of successful implementation [11,12].

For those studies that do provide a detailed account of the intervention, there is inconsistent use of terminology that limits meta-analyses and contributions to science. For example, 'behavioural counselling', 'academic detailing', and 'outreach' can mean very different things according to the group delivering or evaluating the intervention, leaving potential users confused. Having consistent terminology and sufficient information for replication appears to be more problematic for behavioural and organisational interventions than for pharmacological ones. Twenty-six multidisciplinary researchers attending a workshop were presented with a set of behavioural or pharmacological intervention protocols, and asked whether they had sufficient information to be able to deliver them in practice settings. They were less confident about being able to replicate behavioural interventions compared with pharmacological interventions (t = 6.45, p < 0.0001) and judged that they would need more information in order to replicate behavioural interventions (U = 35.5, p = 0.022) [13]. A more detailed protocol description of the intervention did not increase confidence, suggesting that, in this situation at least, more information does not, *per se*, make intervention descriptions easier to interpret and to use for replication.

The lack of attention to providing useful descriptions of behavioural interventions may in part reflect the low investment in this area of research (compared to the investment in pharmacological research); it also may reflect limitations in current scientific practice. Intervention development methods and content are often based on simple, mostly unstated models of human behaviour or, at best, are 'informed' by theory using methods that are tenuous and intuitive rather than systematic [14,15]. This means that each new intervention and each new evaluation occurs in relative isolation, and the opportunity to build an incrementally improving 'technology' of behaviour change is constrained. If a more explicitly theoretical approach to deciding how to design and report interventions were taken, it may be that more effects may be revealed and more understanding of their functional mechanisms gleaned. Arguably, better reporting of interventions that are poorly (and implicitly) conceptualised will not improve the situation. Advantages of using explicit rather than implicit theoretical models include providing a consistent and generalisable framework within which to gather evidence; promoting the understanding of causal mechanisms that both enrich theory and facilitate the development of more effective interventions [16]; and suggestimg moderating variables that would guide the user in adapting the intervention to different patients or population subgroups [4,17]. The extent to which this advantage is realised will depend on the development of more sophisticated methods of applying theory to intervention design and evaluation [18].

### The advantages of reporting interventions better

To implement interventions to provide benefits to the intended populations, the functional components of interventions must be known and clearly described. For example, in pharmacology the active ingredient of aspirin is very different from the active ingredient of statins, and each is known to impact on physiological and pathological outcomes in different ways. To accumulate evidence of outcome effectiveness and of processes of behavioural change, accurate replication of such interventions across multiple studies is required. An analysis of 49 highly cited clinical research studies found that, of 45 claimed to be effective, only 20 (44%) had their findings replicated by subsequent research [19]. Replication requires accurate and detailed reporting of the interventions. Such replication generates scientific knowledge, allows unhelpful or even harmful interventions to be avoided, and provides the detail that allows effective interventions to be subsequently introduced and scaled up to provide population benefits. There is evidence that the more clearly the effective core components of an intervention are known and defined, the more readily the program or practice can be introduced successfully [20-22]. The core intervention components are, by definition, essential to achieving good outcomes for those targeted by the intervention. This is as true for modes of delivery and intervention settings as it is for intervention content. As a simple example, a core component of Multi-systemic Therapy (MST), Homebuilders, and Nurse-Family Partnership (NFP) interventions is that they are delivered in the homes of children [23-25]. It is not MST, Homebuilders, or NFP unless this fundamental feature is present. However, in a large scale attempt to replicate Homebuilders across the United States, many of the replication sites delivered services in their offices, not family homes and, predictably, the outcomes were disappointing [26]. The philosophy and values of Homebuilders were adopted, but the core intervention components were not used. Thus, the specification of effective core intervention components becomes very important to the process of the subsequent introduction of innovations on a scale useful to society and to their evaluation in practice (*e.g*., [4,27,28]).

Knowing the effective core intervention components may allow for more efficient and cost effective introduction of interventions and lead to confident decisions about the non-core components that can be adapted to suit local conditions at a local site. Not knowing the effective core intervention components leads to time and resources wasted in attempting to introduce a variety of non-functional elements. Clear descriptions of core components allow for evaluations of the functions of those procedures. Some specific procedures and sub-components may be difficult and costly to evaluate using randomised group designs (*e.g*., [29]), but within-person or within-organisation research designs offer an efficient way to experimentally determine the function of individual components of evidence-based practices and programs [18,30-34]. For those interventions that are supported by a series of randomized controlled trials (RCTs) that are theoretically and methodologically consistent across studies, Bloom has suggested meta-analytic strategies to take advantage of naturally-occurring variations in RCTs to discern effective components of interventions for different types of participant and setting. Of course, as with any meta-analysis, the results depend on having investigators 'guess right' about the core components for which measures are included.

### Current reporting guidelines

Guidelines for researchers to improve the transparent and accurate reporting of interventions in health research are summarized on the EQUATOR Network website . They include the well-established CONSORT guidelines for reporting evaluation trials, which suggest that evaluators should report 'precise details of interventions [as] actually administered' [35]. The extension of these guidelines to non-pharmacological trials [36], the TREND Statement for the transparent reporting of evaluations with non-randomised designs [37] and the STROBE Statement for strengthening the reporting of observational studies [38] all call for intervention content to be described, as do the SQUIRE guidelines for quality improvement reporting [39,40]. However, it is only recently that attention has begun to be paid, by groups such as the Workgroup for Intervention Development and Evaluation Research (WIDER), to what, or how to, report intervention content and components. Their current recommendations to improve reporting of the content of behaviour change interventions are available at .

### The relationship between post-hoc and ante-hoc description

The reporting guidelines cited above are intended to be used as a post-hoc set of descriptors. However, in order to maximise the scientific advantages inherent in better description, we argue that there needs to be an 'ante-hoc' process that informs the building of the intervention in the first place. This is consistent with the increasing practice of researchers involved in healthcare implementation studies to describe study and intervention protocols in BMC journals such as *Implementation Science*; because there is no formal space limit, intervention materials such as leaflets, brochures, websites, and training schedules can be easily included using facilities such as Additional Files.

## Future developments

### An overall framework for describing important elements of an intervention

Advances in intervention reporting will require greater clarity about both what to report and how to report. Eight characteristics have been identified as essential descriptors in relation to public health interventions [41]: the content or elements of the intervention (techniques), characteristics of those delivering the intervention, characteristics of the recipients, characteristics of the setting (*e.g*., worksite), the mode of delivery (*e.g*., face-to-face), the intensity (*e.g*., contact time), the duration (*e.g*., number sessions over a given period), and adherence to delivery protocols. Adherence is not a characteristic of interventions *per se*, and is outside the focus of this paper, as are indicators of generalisation, such as the RE-AIM elements of reach, effectiveness/efficacy, adoption, implementation, and maintenance [4]). Work towards defining characteristics of intervention designed to improve professional practice and the delivery of effective health services has begun by the Cochrane Effective Practice and Organisation of Care Group . It covers a wide range of characteristics, *e.g*., evidence base, purpose, nature of desired change, format, deliverer, frequency/number of intervention events, duration, and setting. However, neither framework provides a method of reporting intervention content, *i.e*., the component techniques.

Work in the UK has begun to construct a nomenclature of behaviour change techniques. Using inductive and consensus methods, systematic reviews of behaviour change interventions and relevant textbooks have been analysed [14,42]. This has generated a list of 137 separately defined techniques representing different levels of complexity and generality [13], and a 26-item list of techniques demonstrating good inter-rater reliability across raters and behavioural domains [42]. The latter, along with a coding manual of definitions, was inductively generated from systematic reviews of interventions (84 comparisons) using behavioural and/or cognitive techniques, some in combination with social and/or environmental and policy change strategies.

This nomenclature has been used to code interventions in a systematic review of interventions to increase physical activity and healthy eating [43]. This demonstrated that the interventions comprised, on average, six techniques (ranging from one to 14). By combining this analysis with meta-regression, it is possible to analyse the effects of individual techniques and technique combinations within these mainly multifaceted interventions. Using this method, interventions that combined self-monitoring with at least one other technique derived from control theory were significantly more effective than the other interventions, an effect that would have been missed using traditional meta-analyses. A similar approach has been used by Chorpita, Daleiden, and Weisz [44] to code and catalogue common features of evidence-based behavioral interventions. These features should include recipients (demographics), setting, mode of delivery, and key targets (*e.g*., knowledge, skills, and attitudes). This would represent a significant advance on analysing the overall effect size of heterogeneous interventions.

### An agreed set of terms

Because different labels can be used for the same intervention technique, and different techniques may be referred to by the same label, it is imperative that there be a consensual, common language to describe an agreed list of techniques. Just as medicines are described in detail in the British National Formulary (BNF), we need a parsimonious list (nomenclature) of conceptually distinct and defined techniques, with labels that can be reliably used in reporting interventions across discipline and country. This was seen as an important tool for describing interventions (mean rating 4.4 on a scale of zero to five, with five most relevant to needs) in the workshop reported above [13].

### The role of theory

In addition to establishing the core components ('active ingredients') of interventions, progress in developing effective interventions requires an understanding of how interventions work, that is, the mechanisms by which interventions cause behaviour change [45]. This requires clear links between defined intervention techniques and theoretical mechanisms of change. There is increasing recognition that the design of behaviour change interventions should be based on relevant theories [4,16,17,46]. This is partly because such interventions are more likely to contribute to the science of behaviour. Using theory to identify constructs (key concepts in the theory) that are causally related to behaviour, and are therefore appropriate targets for the intervention, can confer a range of benefits including potentially stronger effects [47-49].

Use of theory also leads to evaluations that are more useful in developing theoretical understanding. In the UK, the Medical Research Council's framework for developing and evaluating complex interventions placed theory centrally within the process of intervention evaluation [50]. The usefulness of using theory depends on ensuring that techniques are linked directly to the hypothesized causal process that accounts for change. This allows theory to be used to design interventions and evaluations of interventions to be used to develop theory. Next steps in this area of work are to validate and refine the nomenclature of techniques, and identify underlying theoretical principles to produce a taxonomy with a hierarchically organised internal structure.

## Conclusion

The scientific reporting of complex behaviour change interventions is an idea whose time has come; there is simply no reason not to do this. Journals' space constraints have often limited the publication of detailed descriptions of interventions. However, with the advent of Open Access publishing and the possibility of publishing supplementary material on the web, journals should now require a detailed intervention protocol to be made available as a pre-requisite to the publication of a report of an intervention evaluation. The only argument against this is a commercial one, the desire for some researchers to earn money directly from their research activity. Copyright and intellectual property rights are put forward as reasons for not publishing details of their intervention protocols and manuals. This is an ethical and political issue for the scientific community. Do we want to put science first, with all the benefits it will accrue for humanity, or do we want to go down the road of the pharmacological industry, putting profit before health benefits? The development of the World Wide Web could have become a commercial enterprise, benefitting corporations above the scientific community. Due largely to the ethical principles of its creator, Tim Berners-Lee, the web has been retained for the benefit of the public in the face of considerable corporate pressure. It is our hope that the behavioural science community will collectively value public health over private profit, and co-ordinate their efforts to achieve this.

We welcome *Implementation Science*'s new policy (Appendix) of requiring authors to make, or to have made, available intervention protocols when submitting intervention studies and to report interventions, guided by Davidson *et al*.'s characteristics (see above) and based on the WIDER Recommendations to Improve Reporting of the Content of Behaviour Change Interventions . We also welcome the advice to authors to identify in protocols what they think are prototypical/core elements of interventions, hypothesised mediating mechanisms, and potential moderators. The editorial policy of *Implementation Science *is one step in this direction; seeking agreement from other journals to introduce similar policies will be essential to the strengthening of our science and enhancing the impact of its findings.

## Competing interests

The authors declare that they have no competing interests. ME is Co-Editor in Chief of *Implementation Science*, SM, DF and JMG are members of the Editorial Board of *Implementation Science*. SM is a member of the WIDER Group.

## Authors' contributions

SM conceived the idea for the paper and led the writing. DF, ME and JMG contributed to the writing and commented on all drafts.

## Appendix

### *Implementation Science *editorial policy on describing the content of complex interventions

In order to achieve the benefits discussed in this editorial, authors submitting to *Implementation Science *will be required to provide detailed descriptions of the interventions delivered in their studies.

These are the WIDER Recommendations to Improve Reporting of the Content of Behaviour Change Interventions 

#### 1. Detailed description of interventions in published papers

Authors describing behaviour change intervention (BCI) evaluations should describe: 1) characteristics of those delivering the intervention, 2) characteristics of the recipients (and see Noguchi *et al*., 2007, for unusual but importantly informative detail on participants before and after attrition), 3) the setting (*e.g*., worksite, time, and place of intervention), 4) the mode of delivery (*e.g*., face-to-face), 5) the intensity (*e.g*., contact time), 6) the duration (*e.g*., number of sessions and their spacing over a given period), 7) adherence/fidelity to delivery protocols, and 8) a detailed description of the intervention content provided for each study group.

#### 2. Clarification of assumed change process and design principles

Authors describing BCI evaluations should describe: 1) the intervention development, 2) the change techniques used in the intervention, and 3) the causal processes targeted by these change techniques; all in as much detail as is possible, unless these details are already readily available (*e.g*., in a prior publication).

#### 3. Access to intervention manuals/protocols

At the time of publishing a BCI evaluation report, editors will ask authors to submit protocols or manuals describing BCI evaluations or, alternatively, specify where manuals can be easily and reliably accessed by readers. Such supplementary materials can be made accessible online.

#### 4. Detailed description of active control conditions

Authors describing BCI evaluations should describe the content of active control groups in as much detail as is possible (*e.g*., the techniques used) in a similar manner to the description of the content of the intervention itself.

From 2009 authors will be strongly encouraged to provide this information; from 2011 they will be required to provide it
